# A Field-Based Study of the Magnitude of Risk Factors and Health Habits in Young Volunteers in the Community

**DOI:** 10.7759/cureus.15821

**Published:** 2021-06-22

**Authors:** Turki Alsafrani, Abdulkarim W Abukhodair, Osama M Khojah, Essam I Jastania, Rawan Alamri, Abdulhalim J Kinsara

**Affiliations:** 1 Orthopaedics, King Saud Bin Abdulaziz University for Health Sciences, College of Medicine-Western Region, Ministry of National Guard Health Affairs, Jeddah, SAU; 2 Surgery, King Saud Bin Abdulaziz University for Health Sciences, College of Medicine-Western Region, Ministry of National Guard Health Affairs, Jeddah, SAU; 3 Internal Medicine, King Saud Bin Abdulaziz University for Health Sciences, College of Medicine-Western Region, Ministry of National Guard Health Affairs, Jeddah, SAU; 4 Cardiac Surgery, King Abdulaziz University Hospital, Jeddah, SAU; 5 Cardiology, King Saud Bin Abdulaziz University for Health Sciences, College of Medicine-Western Region, Ministry of National Guard Health Affairs, Jeddah, SAU; 6 Cardiology, King Abdullah International Medical Research Center, Jeddah, SAU

**Keywords:** cigarette smoking, hypertension, kingdom of saudi arabia (ksa), dyslipidemia, diabetes mellitus, young people, community obesity

## Abstract

Objective

A field study is more informative in terms of epidemiological data than a hospital-based study. Undiagnosed risk factors may be discovered in an asymptomatic group. This study aimed to estimate if the community was well informed about the risk factors for coronary artery disease and if that affected the prevalence and the anthropometric among those who participated in the study.

Materials and methods

A cross-sectional study was conducted, using a consecutive sampling technique. Individuals were interviewed in terms of the risk factors and clinical signs and symptoms. The anthropometric measurements were done on-site to identify asymptomatic risk factors. The survey was utilized to increase the awareness among the participants.

Results

In total, 193 individuals participated in this study. The mean age of the sample was 36.3 ± 12.4 years, with 53% male. Smoking was the most frequent risk factor (31.6%), followed by dyslipidemia (22.5%), hypertension (16.6%), and diabetes mellitus (14.5%). Almost half of the sample participated in sports for one to two hours per week (40%). Almost all consumed fast food at least once a week, and 16.6% consumed fast food more than four times a week. The average systolic blood pressure was 129.41 ± 22.5 mmHg and the average body mass index (BMI) 27.6 ± 7.2 kg/m2.

Conclusion

Dyslipidemia was the most prevalent risk factor. Hypertension and diabetes mellitus are on top of the risk factor pyramid in commonality. An early diagnosis is important to decrease the incidence of cardiovascular disease. The consumption of fast food and obesity are relatively high and require educational interventions and more available healthy food. Screening through social media and primary health care centers may avert a negative outcome.

## Introduction

The World Health Organization stated that in 2019, mortality due to ischaemic heart disease (IHD) increased by more than 1.2 million in upper-middle-income countries. IHD and stroke were the two major causes of death [1]. Traditional risk factors are still prevalent, underdiagnosed, or under-treated. In Saudi Arabia, a study reported that a high-fat diet was the most prevalent risk factor (73.4%), followed by physical inactivity (57.9%). It should be noted that this study was conducted with medical students, a well-educated group [2]. Another study estimated the prevalence of the conventional risk factors, including hypertension (HTN), dyslipidemia, diabetes mellitus (DM), and smoking, in coronary artery disease (CAD) patients as 84.6% in women and 80% in men [3]. To reduce the burden of cardiovascular disease, we should determine the risk factors in the community and prevent their progression. An updated and inclusive prevalence study in the community measures the risk factors and reduces the knowledge deficit. This article was previously presented as a meeting abstract at the ESC Acute CardioVascular Care 2021 annual scientific meeting on March 13, 2021.

## Materials and methods

Study setting and participants

We conducted a cross-sectional study to measure the prevalence of CAD-related risk factors in the community of the Western Region of Saudi Arabia. A non-probability consecutive sampling technique was used. The questionnaire was partly self-administered and completed by a trained data collector. Participants who were 18 years and older were included. Informed consent was obtained.

Study variables

Demographic information was collected and dichotomous questions were used to explore current chronic diseases, current diagnoses of HTN, DM, dyslipidemia, and chronic heart failure. Questions related to lifestyle risk included hours of activities per week, the type of activities, fast-food meals consumed per week, and smoking. The second section was the measurement of weight, height, systolic and diastolic blood pressure in both arms. The BMI was classified according to WHO classes.

Statistical methods and ethical considerations

The quantitative variables are presented as mean ± standard deviation and the qualitative variables as frequency and percentage. The chi-square test or Fisher’s exact test, as appropriate, assessed the association between two categorical variables. The statistical analyses were performed with IBM Statistical Software for Social Sciences (SPSS Statistics) for Windows, version 25 (IBM Corp., Armonk, N.Y., USA). Ethical approval was obtained from the Institution Review Board of King Abdullah International Medical Research Center.

## Results

In total, 193 mall visitors volunteered to participate in the study. More than half were male (n=97, 53.9%), and the mean age was 36.1 ± 12.4 years (95% CI = 34.3 to 38.1 years). Most of the participants were younger than 39 years (62%), and overweight or obese (65.4%). The mean systolic blood pressure was 129.41 ± 22.5 mmHg (95% CI = 126.1 to 132.6 mmHg) (Table 1).

**Table 1:** Demographic information of the sample

Demographics		n	%
Gender	Male	97	53.9
	Female	83	46.1
Age (y)	Below than 29 Years	58	32.4
	30-39 years	53	29.6
	40-49 years	37	20.7
	Above than 50 years	31	17.3
BMI (kg/m^2^)	Underweight	15	8.1
	Normal weight	49	26.5
	Overweight	57	30.8
	Obese	64	34.6
Blood pressure (mmHg) (mean ± SD)	129.4 ± 22.5 mmHg		

The highest reported risk factor in the sample was dyslipidemia (22.5%), followed by HTN (16.6%), DM (14.5%), hypothyroidism (4.3%), and congestive cardiac failure (2.6%). The majority of the group who had DM were above 50 years old (60%) and dyslipidemia was diagnosed in 43.9%. The majority of the group (78.6%) with previously diagnosed HTN were above 40 years old. The highest proportion of the overweight participants were 30-39 years old. There was a significant association between age and comorbidities (p > 0.001) (Table 2).

**Table 2:** Association between age groups and comorbidities *= chi-square test **= Fisher’s exact test

Comorbidities		Below 29 Years (%)	30-39 years (%)	40-49 years (%)	Above 50 years (%)	Total	p-value
Diabetes Mellitus	Yes	1 (4)	4 (16)	5 (20)	15 (60)	25	<0.001*
	No	57 (37)	49 (31.8)	32 (20.8)	16 (10.4)	154	
Hypertension	Yes	1 (3.6)	5 (17.9)	10 (35.7)	12 (42.9)	28	<0.001*
	No	57 (37.7)	48 (31.8)	27 (17.9)	19 (12.6)	151	
Congestive Heart Failure	Yes	1 (20)	1 (20)	1 (20)	2 (40)	5	0.598**
	No	56 (32.4)	52 (30.1)	36 (20.8)	29 (16.8)	173	
Dyslipidemia	Yes	1 (2.4)	12 (29.3)	10 (24.4)	18 (43.9)	41	<0.001*
	No	55 (41.4)	40 (30.1)	26 (19.5)	12 (9)	133	
Hypothyroidism	Yes	1 (12.5)	3 (37.5)	1 (12.5)	3 (37.5)	8	0.290**
	No	56 (33.5)	50 (29.9)	35 (21)	26 (15.6)	167	
Body Mass Index	Underweight	12 (80)	2 (13.3)	1 (6.7)	0 (0)	15	<0.001*
	Normal weight	23 (50)	13 (28.3)	5 (10.9)	5 (10.9)	46	
	Overweight	10 (18.2)	22 (40)	14 (25.5)	9 (16.4)	55	
	Obese	10 (17.5)	15 (26.3)	16 (28.1)	16 (28.1)	57	

A third of the sample smoked (31.6%), with the majority smoking cigarettes (89.2%). Fast-food meal consumption was high, with four or more fast-food meals per week (24.6%) and a third of the sample consumed one meal per week (34.3%). The fast-food consumption was significantly higher in the younger age group (p = 0.037), which was concerning as it was accompanied by physical inactivity. In the younger age group, a third were physically inactivity (28.3%) and 39.8% were active one to two hours per week. For the group that exercised, the main activity was walking (64.1%), though for a short period (Table 3). 

**Table 3:** Association between age groups and lifestyle habits *= chi-square test **= Fisher’s exact test

Habits		Below 29 Years (%)	30-39 years (%)	40-49 years (%)	Above 50 years (%)	Total	p-value
Smoking	Yes	20 (35.1)	21 (36.8)	11 (19.3)	5 (8.8)	57	0.149*
	No	37 (30.6)	32 (26.4)	26 (21.5)	26 (21.6)	121	
Smoking Method	Cigarettes	16 (31.4)	20 (39.2)	10 (19.6)	5 (9.8)	51	0.368**
	E-cigarettes	4 (66.7)	1 (16.7)	1 (16.7)	0 (0)	6	
Fast-Food Meals Consumption	No Meals	0 (0)	1 (100)	0 (0)	0 (0)	1	0.037*
	One Meal	13 (22.4)	13 (22.4)	16 (27.6)	16 (27.6)	58	
	Two Meals	14 (40)	11 (31.4)	6 (17.1)	4 (11.4)	35	
	Three Meals	14 (53.8)	8 (30.8)	1 (3.8)	3 (11.5)	26	
	More Than 4 Meals	15 (35.7)	16 (38.1)	7 (16.7)	4 (9.5)	42	
Hours of Exercise per Week	No Exercises	14 (30.4)	15 (32.6)	10 (21.7)	7 (15.2)	46	0.442*
	1-2 hours	23 (31.9)	20 (27.8)	14 (19.4)	15 (20.8)	72	
	3-4 hours	8 (40)	2 (10)	4 (20)	6 (30)	20	
	5 hours and more	13 (32.5)	15 (37.5)	9 (22.5)	3 (7.5)	40	
Type of Exercises	Walking	23 (27.1)	22 (25.9)	20 (23.5)	20 (23.5)	85	0.427*
	Running	5 (35.7)	4 (28.6)	3 (21.4)	2 (14.3)	14	
	Weightlifting	7 (43.8)	6 (37.5)	3 (18.8)	0 (0)	16	
	Swimming	6 (46.2)	1 (7.7)	4 (30.8)	2 (15.4)	13	
	Others	3 (50)	2 (33.3)	1 (16.7)	0 (0)	6	

## Discussion

Diabetes mellitus, HTN, and dyslipidemia were more prevalent in the older age groups. Although the study highlights a high rate of risk factors, the prevalence of the risk factors is lower than reported in previous studies. In the current study, dyslipidemia was 22.5%, compared to 45% reported in 2007 [4]. HTN prevalence was 16.6% compared to 26.1% in 2000 [5], and DM 14.5% compared to 23.7% in 2004 [6]. However, the prevalence of DM, HTN, and dyslipidemia in the population is high compared with other global populations [7].

The mean blood pressure is higher than recommended in current guidelines [8]. The mean blood pressure and BMI also increased [9]. Increased awareness of physical activity was noted but the hours of physical activity were less than the recommended guideline [10]. In 2007, the prevalence of inactivity was 96.1% compared with 28.3% in the current study [11]. The challenge related to physical inactivity is global, with a higher prevalence reported in both developing and developed countries [12-14].

The consumption of fast food in the younger age group is alarming and much higher compared to other populations [14, 15]. The efficient use of social media and the mandatory display of the calories of different food items may support a lifestyle change.

The younger age groups had a higher proportion of cigarette smokers, slightly better than reported in a small study in 2003, which was 52.3% [16]. The decline in smoking in developing countries is not comparable to many developed countries and smoking cessation requires more attention [17].

Based on the results, more age-targeted preventive measures such as educational programs for weight loss, the nutritional values of fast-food meals, programs for smoking cessation, and screening programs for dyslipidemia and HTN are required. The research findings increase the growing body of knowledge about the risk factors in the community and the understanding of the most effective preventive strategies.

The limitations of the study were the cross-sectional design, which may lead to bias, as well as the low sample size. The COVID-19 social distancing limited the expansion. However, a field study is known to require extra effort, manpower, and funding, which were not available for our study.

## Conclusions

Dyslipidemia was the most prevalent risk factor, followed by hypertension. Unhealthy lifestyle habits, including smoking, and the high consumption of fast-food meals are prevalent in the younger age groups, resulting in higher blood pressure and BMI than recommended in the current guidelines. Although the sample was among young patients, the gap in awareness of risk factors and itemized components of metabolic syndrome was prevalent. We recommend a focused approach to diet and exercise at an early age and beginning at early school. More effort to provide a suitable environment and the use of social media will enhance the success of this approach. Attention to an early screening of metabolic elements is an essential additional component to reduce the risk of cardiovascular disease.
